# Women’s Experience of Facility-Based Childbirth Care and Receipt of an Early Postnatal Check for Herself and Her Newborn in Northwestern Tanzania

**DOI:** 10.3390/ijerph16030481

**Published:** 2019-02-07

**Authors:** Dunstan R. Bishanga, Joseph Massenga, Amasha H. Mwanamsangu, Young-Mi Kim, John George, Ntuli A. Kapologwe, Jeremie Zoungrana, Mary Rwegasira, Adrienne Kols, Kathleen Hill, Marcus J. Rijken, Jelle Stekelenburg

**Affiliations:** 1 Jhpiego Tanzania, Dar es Salaam, Tanzania; joseph.massenga@jhpiego.org (J.M.); amasha.mwanamsangu@jhpiego.org (A.H.M.); jeremie.zoungrana@jhpiego.org (J.Z.); mary.rwegasira@jhpiego.org (M.R.); 2 Department of Health Sciences, Global Health, University Medical Centre Groningen, University of Groningen, 9713 GZ Groningen, The Netherlands; jelle.stekelenburg@online.nl; 3 Jhpiego, Baltimore, MD 21231, USA; young-mi.kim@jhpiego.org (Y.-M.K.); adrienne.kols@jhpiego.org (A.K.); 4 USAID’s Maternal and Child Survival Program/Jhpiego Tanzania, Dar es Salaam, Tanzania; john.george@jhpiego.org; 5 President’s Office—Regional Administration and Local Government, Dodoma, Tanzania; ntuli.kapologwe@tamisemi.go.tz; 6 USAID’s Maternal and Child Survival Program/Jhpiego, Baltimore, MD 21231, USA; Kathleen.Hill@jhpiego.org; 7 Department of Obstetrics and Gynecology, Division of Woman and Baby, University Medical Center Utrecht, 3584 CX Utrecht, The Netherlands; mrijken2@umcutrecht.nl; 8 Julius Global Health, Julius Center for Health Sciences and Primary Care, University Medical Center Utrecht, 3584 CX Utrecht, The Netherlands; 9 Department of Obstetrics and Gynecology, Leeuwarden Medical Centre, 8934 AD Leeuwarden, The Netherlands

**Keywords:** women’s experience of care, respectful care, facility-based childbirth, early postnatal check, disrespect and abuse, Tanzania

## Abstract

Negative experiences of care may act as a deterrent to current and/or future utilization of facility-based health services. To examine the situation in Tanzania, we conducted a sub-analysis of a cross-sectional household survey conducted in April 2016 in the Mara and Kagera regions of Tanzania. The sample included 732 women aged 15–49 years who had given birth in a health facility during the previous two years. Log binomial regression models were used to investigate the association between women’s experiences of care during childbirth and the receipt of early postnatal checks before discharge. Overall, 73.1% of women reported disrespect and abuse, 60.1% were offered a birth companion, 29.1% had a choice of birth position, and 85.5% rated facility cleanliness as good. About half of mothers (46.3%) and newborns (51.4%) received early postnatal checks before discharge. Early postnatal checks for both mothers and newborns were associated with no disrespect and abuse (RR: 1.23 and 1.14, respectively) and facility cleanliness (RR: 1.29 and 1.54, respectively). Early postnatal checks for mothers were also associated with choice of birth position (RR: 1.18). The results suggest that a missed opportunity in providing an early postnatal check is an indication of poor quality of the continuum of care for mothers and newborns. Improved quality of care at one stage can predict better care in subsequent stages.

## 1. Introduction

Maternal and neonatal mortality remains high in Tanzania: 556 maternal deaths per 100,000 live birth were recorded by the 2015–2016 Tanzania Demographic and Health Survey and Malaria Indicator Survey (TDHS-MIS) compared with 454 maternal deaths per 100,000 live births recorded by the 2010 TDHS [1]. Neonatal mortality is also high, at 25 per 1000 live births [1]. Efforts to reduce maternal and newborn morbidity and mortality have emphasized increasing facility-based childbirths with skilled attendants [2]. In Tanzania, the proportion of deliveries that take place at a facility with the assistance of a skilled provider has risen steadily over the past two decades, from 44% in 1999 to 50% in 2010 and 63% in 2015–2016 [3,4]. However, the increase in facility deliveries has not been matched by an anticipated decrease in maternal and newborn deaths. Poor quality of care during childbirth in health facilities, not least in the continuum of care, is probably the most important factor to explain the discrepancy between the observed high coverage of institutional deliveries and stubbornly high maternal and perinatal mortality rates [5].

The first days after childbirth—the early postnatal period—are a critical phase in the lives of mothers and newborn babies [6,7]. Most maternal deaths occur within 48 h of delivery, and more than three-quarters of newborn deaths occur in the first week after birth [8]. However, skilled care throughout pregnancy, labor and delivery, and the early postnatal period can reduce the risk of death or disability for both the mother and baby [9,10]. Postnatal care (PNC) ideally should be provided within 48 hours to all women delivering in a health facility and their newborns [7,11,12]. Multiple studies from Africa and Asia have found that delivering in a facility and/or being attended by a skilled provider during childbirth increases the likelihood that women and babies will receive early PNC [10,13,14,15].

In Tanzania, however, most mothers (66%) and newborns (58%) do not receive the recommended postnatal check within 48 hours after childbirth according to the 2015–2016 TDHS-MIS, despite the increase in facility deliveries [3]. This is far below the national target of 80% of mothers and newborns receiving an early postnatal check by the year 2020 [8]. After a facility delivery, a postnatal check for the mother and her newborn is recommended before discharge [7,16]; this counts as an early postnatal check.

Many practices contribute to women’s experience of care during childbirth, including communication with providers, respectful treatment, letting mothers choose their birth position, and offering a choice of birth companions [16]. Multiple studies from Tanzania have documented lapses in these elements, including clearly disrespectful behavior [17]. In one study, only about half of mothers were given time to ask questions and more than half described disrespectful, impolite, or unhelpful interactions with staff [18]. Another study found that women could not choose their birth positions but instead had to follow the providers’ directives [19]. Other common problems in Tanzania include neglect, abandonment, and discriminatory treatment associated with monetary demands [20,21]. At best, mothers who deliver in health facilities are ambivalent about the quality of care they receive [22].

Negative experiences of care during facility-based childbirth may act as a deterrent to current and/or future utilization of facility-based services [23,24]. They also constitute an important barrier to increasing the utilization of skilled care and improving maternal health outcomes [24,25]. For example, rural Tanzanian women participating in a discrete choice experiment identified provider attitude and drug availability as the most important facility characteristics influencing their choice of a facility delivery. The authors of the study estimated that improving these would lead to a 43% to 88% increase in facility deliveries [26].

The government of Tanzania has prioritized key interventions to improve both coverage and quality of care around childbirth, particularly for regions in the Lake Zone (including Kagera and Mara regions) which lag behind on various reproductive, maternal, newborn, and child health (RMNCH) indicators [27,28]. However, limited information is available on respectful maternity care in Kagera and Mara. At the time of writing this article, there were no studies on the association between respectful care during labor and delivery and early postnatal checks after deliveries at a health facility. To fill this gap, this study explored women’s experience of facility-based childbirth care in the two regions, including disrespect and abuse, choice of birth position, offer of a birth companion, and perceived facility cleanliness. We also examined whether women’s self-reported experience of care during childbirth predicts pre-discharge postnatal checks for mother and baby.

## 2. Materials and Methods

### 2.1. Study Design and Setting

This sub-analysis draws on data from a cross-sectional household survey conducted in April 2016 in the Mara and Kagera regions of the Lake Zone of Tanzania by the Maternal and Child Survival Program (MCSP). MCSP has implemented a comprehensive RMNCH program in these regions together with local stakeholders. The survey collected information on knowledge, practices, and coverage (KPC) related to maternal and newborn health, immunization, malaria, and family planning from 1263 women aged 15–49 years who had given birth during the two years preceding the survey.

### 2.2. Sampling Technique and Sample Size

The household survey employed a two-stage stratified cluster sampling design. The survey calculated the minimum sample size requirements for three main outcomes of interest under the simple random sampling survey design; these outcomes included delivery at a health facility, immunization coverage, and modern contraceptive prevalence rate. Since the survey employed a cluster sampling strategy, we inflated the sample size with a design-effect of 1.5 for adjusting higher inter-cluster correlation in the observations in the two regions, which is similar to the 2015–2016 TDHS-MIS [1]. In cases of non-response or non-availability of respondents, no substitutions were made in order to avoid selection bias by the interviewers; therefore, the sample was adjusted by another 10% inflation factor. More details on the methods are available in a previous article that reported on findings from this survey [29].

**Figure 1:**
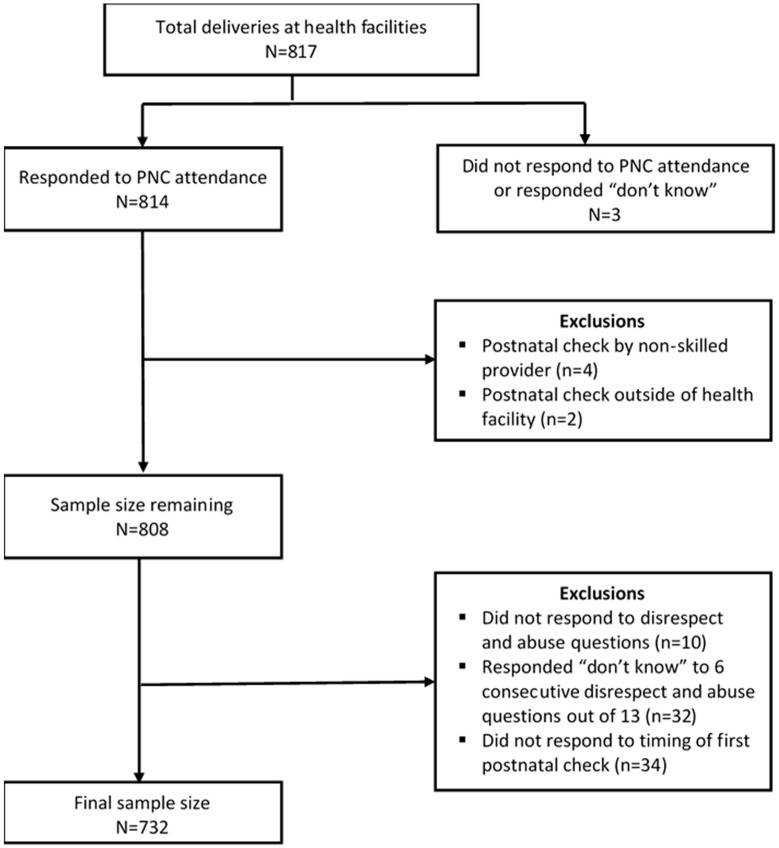
Schematic diagram for exclusions and inclusions to final sample size from KPC household survey in Kagera and Mara.

This sub-analysis extracts data from 817 of the survey respondents who had delivered at a health facility in the past two years. A total of 85 women were excluded from the analysis based on the following criteria (Figure 1): three women did not respond or answered “don’t know” to the postnatal check questions; four women reported receiving a postnatal check from someone other than a health care worker (e.g., a relative or mother-in-law); two women received a postnatal check outside of a health facility; 10 women did not respond to any survey questions on disrespect and abuse; 32 women responded “don’t know” to at least six of the 13 survey questions on disrespect and abuse; and 34 women did not provide information on the timing of their first postnatal check after delivery.

The final sample included in the sub-analysis consisted of 732 women, which is equivalent to over 90% power in detecting the real difference in early postnatal checks among recently delivered women between exposure groups, assuming 44% of women had an early postnatal check and a 10% margin of error.

### 2.3. Measurement of Variables

Early postnatal checks for the mother and newborn were the primary study outcomes for this sub-analysis. These were defined as any health care services given by a professional health worker (doctor/clinician or nurse/midwife) at a health facility prior to discharge and within 48 h of delivery. This was based on information reported by women during the household survey.

The sub-analysis assesses four aspects of women’s experience of care: disrespect and abuse, choice of birth companion, choice of birth position, and facility cleanliness. To assess disrespect and abuse during labor or delivery, the survey asked women if they had undergone any of the following 13 experiences: left alone for a long period of time, left to deliver unassisted/alone, verbally abused, shared a bed with another person during labor, unhappy with the level of privacy, provided with no bed sheet, pinched/slapped/pushed/beaten, subjected to inappropriate touching, discriminated against based on specific patient attributes, denied services due to lack of money, detained in the health facility until money was paid, denied food/drink, or interventions done without being explained/consented. These 13 experiences were developed specifically for the Tanzania survey based on a landscape analytical framework developed by Bowser and Hill in 2010 that described seven manifestations of disrespect and abuse of women during childbirth [25]. This framework has also been used in systematic reviews on disrespect and abuse [30]. Choice of birth companion assesses whether health workers offered the woman an opportunity to have a companion present during labor or delivery. Choice of birth position assesses whether the woman was offered an opportunity to choose a position during childbirth. Facility cleanliness assesses whether the woman was happy with the overall cleanliness of the facility.

### 2.4. Data Collection, Management, and Analysis

The structured questionnaire used to collect data was developed by the Child Survival Health Grants Program and MCSP. RMNCH technical experts based in Tanzania adapted the survey questions to the local context. The questionnaire was then translated into the Kiswahili language and reviewed by local experts who revised some questions to make their meaning clearer in Kiswahili.

We recruited 30 female and male research assistants and trained them on research ethics, the study protocol, household sampling, informed consent, and other data collection procedures. They collected data during face-to-face interviews conducted in Kiswahili. Women’s responses were recorded on password-protected tablets, using the CommCare HQ (Dimagi, Cambridge, MA, USA) mobile data collection platform. Steps were taken to assure data quality by building skip patterns into the survey. In addition, a data manager reviewed the information collected each day and immediately alerted study supervisors to errors that needed to be addressed.

Data management and analyses were performed using STATA 14 (College Station, TX, USA). The primary outcome of the analysis was receiving an early postnatal check for both mother and newborn before discharge from the health facility and within 48 h of delivery. Respondents who reported receiving an early postnatal check were compared with those who did not.

During data analysis, we examined categorical variables as frequencies and percentages and used Pearson’s chi square to test for differences. Fisher’s exact test was applied whenever there were fewer than five participants in one or more categories. We used log binomial regression models to examine the association between women’s experience of care (including disrespect and abuse, birth position, birth companion, and facility cleanliness) and early postnatal checks. The multivariable model included variables that were statistically significant at the 20% level in the bivariate analysis (region, education level, number of children, mode of delivery, cadre who attended the delivery, level of health facility, and number of antenatal care (ANC) visits) and key determinants of public health-related outcomes such as age, even though they were not significant in the bivariate analysis [31]. Both interaction and multicollinearity were assessed in the final fitted model while clustering at the level of the enumeration areas (primary sampling unit) was accounted for by the use of generalized estimating equations for the multivariable regression. Results were presented as risk ratios with corresponding 95% confidence interval as well as *p*-values.

### 2.5. Ethical Considerations

Research assistants collected oral consent from all study participants. First, they read the consent form aloud in Kiswahili and answered any questions the woman had. If a woman agreed to participate, the research assistant wrote down her identification number (retrieved from a pre-assigned list) on the consent form and signed it to certify that the woman gave her consent. A parent’s or guardian’s assent was also required for unmarried mothers aged 15–17 to participate in the study. To protect women’s privacy, interviews were conducted in an area of the home where no one could see or overhear what was said. This study was approved by the Johns Hopkins School of Public Health Institutional Review Board (IRB No. 00005931) and the Medical Research Coordinating Committee of the National Institute for Medical Research of Tanzania (No. NIMR/HQ/R.8a/ Vol.IX/2131).

## 3. Results

### 3.1. Characteristics of the Study Population

The analysis includes data from 732 mothers who gave birth at health facilities. More than half were from Kagera region (55.9%), nearly half were aged 15–24 (44.5%), and about two-thirds (69.7%) had completed primary education (Table 1). A majority of them were married (85.5%) and had two or more children (74%). Over half (58.9%) had made four or more ANC visits, 93.4% had a normal vaginal delivery, and 74.3% were attended by nurse/midwives during delivery.

### 3.2. Prevalence of Disrespect and Abuse

**Figure 2:**
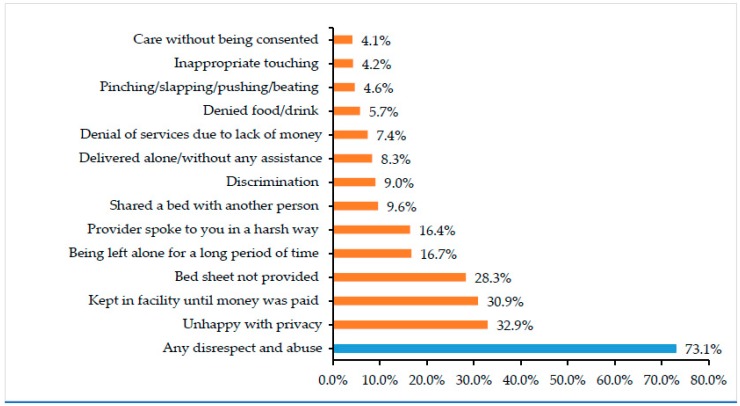
Frequency of specific forms of disrespect and abuse reported by women who gave birth in health facilities in Kagera and Mara regions within the past 2 years (*n* = 732).

Overall, 73.1% of women reported at least one form of disrespect and abuse during delivery (Table 1). Most commonly reported were “unhappy with privacy” (32.9%), “detained in facility until paid money” (30.9%), and “bedsheet not provided” (28.3%) (Figure 2).

A significantly higher proportion of women from Mara than Kagera (81.1% vs. 66.8%) reported some form of disrespect and abuse, as did women who delivered at a health center/dispensary compared with women who delivered at a hospital (76.6% vs. 68.9%). There were no significant differences in reports of disrespect and abuse by women’s age, education, marital status, number of children, wealth quintile, number of ANC visits, mode of delivery, or cadre of birth attendant.

### 3.3. Coverage of Early Postnatal Checks for Women and Newborns

Overall, 46.3% of mothers reported receiving an early postnatal check. Significantly higher proportions of women who lived in Kagera (50.6%), made four or more ANC visits (48.7%), had a caesarean delivery (66.7%), or were attended by a doctor or clinician during the birth (58.3%) received an early postnatal check (Table 2).

Around half (51.4%) of newborns received an early postnatal check before discharge from the facility, and there were significant differences by number of ANC visits and mode of delivery. A higher proportion of babies born to mothers who made at least four ANC visits received an early postnatal check, compared with mothers who did not receive any ANC (54.9% vs. 33%). Over three-quarters (73.8%) of babies delivered by caesarean section received an early postnatal check compared with half (50%) of babies delivered vaginally.

### 3.4. Women’s Experience of Care during Labor and Delivery and Its Association with Early Postnatal Checks

As noted above, 73.1% of women experienced some form of disrespect and abuse during labor and delivery. However, many women also reported positive experiences: 60.1% were offered a choice of birth companion during labor and delivery, 29.1% were offered their choice of birth position, and 85.5% rated facility cleanliness as good.

A bivariate analysis found that all four elements of women’s experience of care were significantly associated with an early postnatal check for the mother, while two elements were associated with an early postnatal check for the newborn: disrespect and abuse and perceived facility cleanliness (Table 3).

### 3.5. Multivariable Log Binomial Regression

The multivariable analysis controlled for region, mother’s age, women’s education, number of children, mode of delivery, cadre of provider attending the delivery, the type of health facility, and the number of ANC visits. After controlling for these factors, early postnatal checks for women were associated with three elements of experience of care (Table 4). Women reporting no disrespect or abuse were 58% more likely to receive an early postnatal check (RR: 1.23, 95% CI: 1.05–2.12). Women offered a choice of birth position were 18% more likely to receive an early postnatal check (RR: 1.18, 95% CI: 1.02–2.23). And women who said the facility was clean were 29% more likely to receive an early postnatal check (RR: 1.29, 95% CI: 1.08–2.84). In contrast, two components of care were associated with early postnatal checks for newborns after controlling for confounders in the multivariable analysis. Newborns were 14% more likely to receive an early postnatal check if their mother reported no disrespect or abuse (RR: 1.14, 95% CI: 1.02–1.92), and they were 54% more likely to receive an early postnatal check if their mother perceived facility cleanliness to be good (RR: 1.54, 95% CI: 1.21–4.12).

Other factors that were associated with early postnatal checks for mothers included women attending four or more ANC visits, giving birth by caesarean section, and giving birth under the care of the clinician/doctor. Postnatal checks for the baby were associated with two other factors: a mother who attended four or more ANC visits and birth by caesarean section.

## 4. Discussion

This study examined two different dimensions of the quality of care at health facilities in two regions of the Lake Zone of Tanzania: first, women’s experiences of care during labor and delivery and, second, coverage of early postnatal checks before women and newborns are discharged. Women’s reports, rather than observations, formed the basis for all measures so the study captures women’s perspectives on the quality of care. Perceived quality of care is important because it affects whether women continue to utilize health services themselves or recommend those services to others [32]. For example, studies have linked women’s experience of care with utilization of PNC [33] and giving birth at a facility in future pregnancies [23,34,35]. A qualitative study in Tanzania revealed that women opt for non-confrontational strategies when they experience disrespect and abuse during childbirth; they simply refrain from using services in future, “voting by their feet” [36].

### 4.1. Women’s Experience of Care

The findings reveal gaps in several elements of care, including high levels of disrespect and abuse, women’s limited control over the choice of birth position and the presence of a birth companion, and deficiencies in facility cleanliness. Almost three-quarters of women in this study reported some form of disrespect and abuse during labor and delivery. Commonly reported problems were both structural (lack of privacy, detainment, and no bed sheet) and personal in nature (abandonment, verbal abuse, unassisted delivery, and physical abuse). The types of disrespect and abuse observed are similar to those found by studies in other parts of Tanzania and other low-income countries [20,21,37,38,39].

The overall extent of disrespect and abuse found in Kagera and Mara is similar to the 70% prevalence found by a study in Dar es Salaam [21] despite the difference in setting; Kagera and Mara are largely rural while Dar es Salaam is a major city. Although health facilities in Dar es Salaam have more equipment, better infrastructure, and a larger and likely more qualified staff, it is possible that fatigue from a heavy client load may contribute to lack of respect in providing maternity care, as has been reported in Malawi [40] and Guinea [41]. In contrast, community interviews in rural northeastern Tanzania found a much lower rate of disrespect and abuse, with just 28% of women reporting at least one form of disrespect and abuse [20]. Regional variations in social norms affecting client-provider interactions offer one possible explanation for this kind of wide variation regarding these disrespectful and abusive practices within the same country, governed by the same health care policies; similar variations have been documented in Ethiopia [42]. In this study, women in Mara were more likely to experience disrespect and abuse than those in Kagera. Mara is known to have more rigid gender norms, which are also manifested in higher rates of female circumcision than in Kagera (32% versus less than 1%) [1]. This implies that the extent of disrespect and abuse during labor and delivery depends not only on working conditions and other health sector factors, but also on social and cultural norms which may lower women’s expectations when receiving care and contribute to the normalization of poor care [43]. Accordingly, high rates of disrespect and abuse call for interventions that can transform rigid gender norms, raise the status of women, and create an informed community that treats women with dignity in all aspects of their lives, including heath care.

Respectful maternity care means more than eliminating disrespect and abuse. It also includes positive practices that empower women to make choices regarding labor and delivery. In this study, less than 30% of women had the opportunity to choose their preferred birth position. Flexibility regarding birth position is one of the most poorly practiced components of respectful maternity care in Africa. For example, other studies have found that providers in Ethiopia allowed only one in five women to choose their position during childbirth [32], while over nine in 10 providers in Malawi did not ask women about their preferred birth position [44]. The supine position is routinely used during childbirth in Tanzania, in part because women are not aware of alternative positions [19]. As Lugina and colleagues noted in their recommendations, changing this practice will require a change in providers’ attitude as well as informing women about alternative birth positions [19].

Providers in Kagera and Mara were more likely to give women an opportunity to choose a birth companion, with three out of five women reporting this practice. This is higher than the rate reported by Sethi et al. in Malawi, where less than one in five women were encouraged to have a companion during childbirth [44]. The problem is that although most health facilities in Tanzania encourage a relative or spouse to accompany the woman during labor at a health facility, the companion is not usually allowed into the labor and delivery room, as the infrastructure in most labor rooms does not provide for visual privacy to the other women in labor. Instead, the companion may be asked to help with other tasks, such as helping follow up the results of investigations from the laboratory (usually within the facility premises) or bringing in food. These kinds of restrictions on birth companions have also been reported in other low- and middle-income countries [37,45]. This is unfortunate, as the literature has shown that the presence of a birth companion exerts a positive influence on the quality of care offered to a laboring woman, including respectful treatment by providers—but only if the birth companion is physically present while care is being provided [36,46,47,48]. To fully implement the practice of birth companions in Tanzania, changes would be needed in the physical structure of labor rooms to offer women more privacy, as well as in the policies and protocols that govern labor and delivery and in education for both providers and families.

The survey also asked mothers whether the level of cleanliness at the facility was good. One in seven women were not satisfied with the facility’s cleanliness. While not a direct measure of respectful care, the cleanliness of the facility does impact patients’ experience of health care. Cleanliness is a proxy indicator for quality of care and institutional readiness to provide good quality services. It has been established that women are likely to equate a facility’s cleanliness with the quality of services provided [49], and this does in fact seem to be the case in this study. Hence, quality of care interventions should also focus on structural aspects of the facility, including cleanliness.

### 4.2. Early Postnatal Checks

We found that about half of mothers and newborns are discharged from a health facility after childbirth without receiving postnatal checks, which is an alarming indicator of suboptimal care. This demands urgent attention by program managers and policy makers. While these results, which only reflect facility-based births, are better than national figures for Tanzania, which include home births, the gap is not as huge as expected. Nationally, two-thirds of mothers (66%) and over half of newborns (58%) do not receive a postnatal check within 48 h following delivery, whether as part of pre-discharge care following a facility delivery, a home visit, or a follow-up facility visit [3].

Early PNC for both mother and baby is a key strategy in saving lives of mothers and newborns [8,16,50,51]. A study from Cambodia documented that not receiving PNC is a key contributor to mothers and newborns discontinuing from the continuum of care [52]. The study findings suggest that missed opportunities to provide PNC may explain why the increasing coverage of institutional deliveries in Tanzania, now at 63%, has not had as much impact on maternal and newborn morbidity and mortality as hoped. As has been reported in other studies, however, this analysis found that early postnatal checks for both mother and newborn were more likely if mothers had made at least four ANC visits during pregnancy and if they gave birth by caesarean section [53,54,55]. Thus, promoting ANC services may have a positive impact on PNC.

Our analysis went beyond previous studies to examine the relationship between women’s experience of care and early postnatal checks. Notably, receiving an early postnatal check was associated with the absence of disrespect and abuse, being offered a choice of birth position, and facility cleanliness; early postnatal checks for newborns were also associated the absence of disrespect and abuse and facility cleanliness. These findings suggest that missed opportunities to provide PNC to mothers and newborns are an indicator of poor quality of labor and delivery services more generally. Recommendations to increase utilization of PNC services have focused on getting women to seek PNC as early as possible [6] and increasing the coverage of institutional deliveries has been considered an effective way to accomplish this [15].

However, our findings indicate that the physical presence of women and newborns at a health facility is not sufficient to assure that they receive PNC. Thus, interventions are needed to ensure that the health system provides PNC to all women and newborns following delivery in a facility. This could be achieved by making care protocols and job aids available at service delivery points, equipping health care workers with correct knowledge and skills, and building quality improvement and accountability systems among health providers, while also making clients aware of what services to expect at various stages of maternity care. Accordingly, efforts to improve coverage of immediate PNC should consider improving the quality of care along the continuum, including the experience of care for women during labor and delivery [10,13].

### 4.3. Strengths and Limitations

The strengths of this study include use of a stratified cluster sampling technique, large sample size, and household-based survey. The study also makes a unique contribution by establishing an association between women’s experience of care during facility-based childbirth and early postnatal checks for mothers and babies, which is the first of its kind in Tanzania. Since this study was not specifically designed to assess experience of care, components of experience of care used in the analysis are not exhaustive. Behaviors and practices, including postnatal checks, were reported by women but not observed or otherwise verified; nor did the study include the providers’ perspective. Thus, there was no direct investigation of hospital actions. Although we controlled for confounders by applying multivariate log binomial regression, the findings demonstrate only associations with no causal inferences. In addition, the findings only apply to the two regions of Kagera and Mara and should not be generalized to the whole of Tanzania.

## 5. Conclusions

Although Tanzania has prioritized improvements in maternal and newborn outcomes around childbirth, progress has been slow. This study assessed the association between women’s experience of facility-based childbirth care and coverage of early postnatal checks for mothers and newborns in Kagera and Mara regions. Our results demonstrate that what a woman experiences during childbirth predicts whether she and her baby receive an early postnatal check. This suggests that maternity care is a continuum of services, where better care at one stage can predict better care in the following stages. Thus, to increase coverage of timely PNC for mothers and newborns, health facilities should ensure that mothers experience good care during labor and delivery. The study team recommends that government agencies create policies and care protocols that incorporate RMNCH clients’ experience of care as a key component in the quality improvement process. Program designers and implementers should consider interventions that focus on improving women’s experience throughout the continuum of antenatal, intrapartum, and postnatal care and ensuring that there is respectful and human-centered maternity care.

## Figures and Tables

**Table 1:** Characteristics of survey respondents and percent who reported any disrespect and abuse during childbirth, by sociodemographic characteristics and services received (*n* = 732).

Sociodemographic and Service Delivery Characteristics	Distribution of Respondents	Percent of Women Who Reported Any Disrespect and Abuse during Facility Childbirth	
	*n* (%)	*n* (%)	*p*-Value
Total	732 (100)	535 (73.1)	
Region			<0.001
Kagera	409 (55.9)	273 (66.8)	
Mara	323 (44.1)	262 (81.1)	
Age (years)			0.732
15–24	326 (44.5)	243 (74.5)	
25–34	309 (42.2)	222 (71.8)	
35+	97 (13.3)	70 (72.2)	
Education level			0.631
No formal education	132 (18.0)	100 (75.8)	
Primary education	510 (69.7)	372 (72.9)	
Secondary education	90 (12.3)	63 (70.0)	
Marital status			0.077
In union	626 (85.5)	465 (74.3)	
Not in union	106 (14.5)	70 (66.0)	
Number of children			0.685
1	190 (26.0)	141 (74.2)	
2+	542 (74.0)	394 (72.7)	
Wealth quintile			0.698
Lowest quintile	147 (20.6)	109 (74.2)	
Lower quintile	149 (20.9)	115 (77.2)	
Middle quintile	132 (18.5)	95 (72.0)	
Higher quintile	144 (20.2)	103 (71.5)	
Highest quintile	141 (19.8)	99 (70.2)	
Number of ANC visits			0.174
None	33 (4.5)	25 (75.8)	
1–3	268 (36.6)	206 (76.9)	
4+	431 (58.9)	304 (70.5)	
Type of facility where the delivery took place			0.021
Hospital	335 (45.8)	231 (68.9)	
Health center/dispensary	397 (54.2)	304 (76.6)	
Mode of delivery			0.483
Vaginal	684 (93.4)	502 (73.4)	
Caesarean section	48 (6.6)	33 (68.7)	
Cadre of provider attending delivery			0.076
Clinician (doctor)	175 (23.9)	117 (84.6)	
Nurse/midwife	544 (74.3)	407 (66.9)	
Other (medical attendant)	13 (1.8)	11 (74.8)	

**Table 2:** Bivariate log binomial regression of mothers and newborns who received an early postnatal check, by sociodemographic and service delivery characteristics (*n* = 732).

Sociodemographic and Service Delivery Characteristics	Women Reported An Early Postnatal Check for Themselves			Women Reporting An Early Postnatal Check for Their Baby		
	*n* (%)	RR [95% CI]	*p*-Value	*n* (%)	RR [95% CI]	*p*-Value
Overall	339 (46.3)			358 (51.4)		
Region						
Kagera	207 (50.6)	1.0		205 (52.7)	1.0	
Mara	132 (40.9)	0.81 [0.68–0.95]	0.010	153 (49.8)	0.94 [0.82–1.09]	0.455
Age (years)						
15–24	160 (49.1)	1.0		166 (53.7)	1.0	
25–34	135 (43.7)	0.89 [0.75–1.05]	0.175	144 (49.2)	0.91 [0.78–1.06]	0.263
35+	44 (45.4)	0.92 [0.72–1.18]	0.528	48 (51.1)	0.95 [0.76–1.183]	0.656
Education level						
No formal education	51 (38.6)	1.0		57 (45.6)	1.0	
Primary education	249 (48.8)	1.26 [1.01–1.59]	0.039	259 (53.2)	1.176 [0.95–1.44]	0.149
Secondary education	39 (43.3)	1.121 [0.81–1.54]	0.481	42 (50.0)	1.10 [0.82–1.46]	0.529
Marital status						
In union	287 (45.9)	1.0		309 (51.5)	1.0	
Not in union	52 (49.1)	1.07 [0.86–1.32]	0.531	49 (51.0)	0.99 [0.80–1.22]	0.934
Number of children						
1	96 (50.5)	1.0		101 (56.1)	1.0	
2+	542 (44.8)	0.88 [0.75–1.05]	0.165	257 (49.8)	0.89 [0.76–1.04]	0.133
Wealth quintile						
Lowest quintile	60 (40.8)	1.0		64 (46.0)	1.0	
Lower quintile	70 (47.0)	1.15 [0.89–1.49]	0.287	80 (54.4)	1.18 [0.94–1.49]	0.160
Middle quintile	60 (45.5)	1.11 [0.85–1.46]	0.434	67 (53.2)	1.15 [0.91–1.47]	0.246
High quintile	65 (50.7)	1.24 [0.96–1.60]	0.093	71 (52.2)	1.13 [0.89–1.44]	0.308
Highest quintile	65 (46.1)	1.134 [0.87–1.47]	0.366	67 (50.4)	1.09 [0.85–1.40]	0.475
Number of ANC visits						
None	9 (27.3)	1.0		11 (33.3)	1.0	
1–3	120 (44.8)	1.64 [0.92–2.91]	0.090	121 (48.2)	1.45 [0.88–2.38]	0.147
4+	210 (48.7)	1.77 [1.01–3.14]	0.044	226 (54.9)	1.65 [1.07–2.69]	0.026
Type of facility where delivery took place						
Hospital	157 (46.9)	1.0		168 (53.2)	1.0	
Health center/dispensary	182 (45.8)	0.98 [0.84–1.14]	0.782	190 (50.0)	0.94 [0.81–1.09]	0.404
Mode of delivery						
Vaginal	307 (44.9)	1.0		327 (50.0)	1.0	
Caesarean section	32 (66.7)	1.49 [1.20–1.84]	<0.001	31 (73.8)	1.48 [1.21–1.80]	<0.001
Cadre of provider attending delivery						
Clinician (doctor)	102 (58.3)	1.0		83 (50.6)	1.0	
Midwife or nurse	235 (43.2)	0.74 [0.63–0.86]	0.012	270 (51.8)	1.03 [0.864–1.22]	0.788
Other (medical attendant)	2 (15.4)	0.26 [0.07–0.95]	<0.001	5 (45.5)	0.891 [0.46–1.74]	0.751

**Table 3:** Bivariate log binomial regression of early postnatal checks for mothers and newborns, by women’s experience of care during childbirth and perception of facility cleanliness (*n* = 732).

Experience of Care	Overall *n* (%)	Women Who Had Early Postnatal Check			Women Whose Baby Had Early Postnatal Check		
		*n* (%)	RR [95% CI]	*p*-Value	*n* (%)	RR [95% CI]	*p*-Value
Experienced any disrespect and abuse							
No	197 (26.9)	110 (55.8)	1.34 [1.11–2.01]	0.001	109 (57.6)	1.21 [1.02–1.69]	0.041
Yes	535 (73.1)	229 (42.8)	1.0		249 (49.6)	1.0	
Offered opportunity to have a companion during labor and delivery							
No	292 (39.9)	114 (42.7)	1.0		130 (51.8)	1.0	
Yes	440 (60.1)	225 (48.4)	1.13 [0.97–1.84]	0.143	228 (51.2)	0.99 [0.78–1.39]	0.896
Offered choice of birth position							
No	519 (70.9)	225 (43.4)	1.0		246 (49.7)	1.0	
Yes	213 (29.1)	114 (53.5)	1.23 [1.04–1.86]	0.009	112 (55.7)	1.11 [0.97–1.81]	0.154
Perceived facility to have good cleanliness							
No	106 (14.5)	35 (33.0)	1.0		32 (32.0)	1.0	
Yes	626 (85.5)	304 (48.6)	1.54 [1.54–3.01]	0.002	326 (54.7)	1.62 [1.70–3.71]	<0.001

RR: risk ratio; CI: confidence interval.

**Table 4:** Multivariable log binomial regression of early postnatal checks for mothers and newborns, by women’s experience of care during childbirth and perception of facility cleanliness (*n* = 732).

Experience of Care and Characteristics	Women Who Had Early Postnatal Check		Women Whose Baby Had Early Postnatal Check	
	aRR [95% CI]	*p*-Value	aRR [95% CI]	*p*-Value
Experienced any disrespect and abuse				
No	1.23 [1.05–2.12]	0.014	1.14 [1.02–1.92]	0.022
Yes	1.0		1.0	
Offered opportunity to have a companion during labor and delivery				
No	1.0		1.0	
Yes	1.04 [0.63–1.65]	0.638	0.91 [0.75–1.23]	0.338
Offered choice of birth position				
No	1.0		1.0	
Yes	1.18 [1.02–2.23]	0.043	1.12 [0.93–1.84]	0.196
Perceived facility to have good cleanliness				
No	1.0		1.0	
Yes	1.29 [1.08–2.84]	0.018	1.54 [1.21–4.12]	<0.001
Region				
Kagera	1.0		1.0	
Mara	0.84 [0.57–1.06]	0.053	0.94 [0.73–1.34]	0.555
Age (years)				
15–24	1.0		1.0	
25–34	0.91 [0.66–1.33]	0.408	0.91 [0.71–1.42]	0.576
35+	0.95 [0.89–1.66]	0.732	0.98 [0.67–1.69]	0.866
Education level				
No formal education	1.0		1.0	
Primary education	1.17 [0.98–2.67]	0.096	1.14 [0.90–1.92]	0.345
Secondary education	1.01 [0.56–1.86]	0.972	1.00 [0.64–1.93]	0.993
Number of children				
1	1.0		1.0	
2+	0.96 [0.65–1.48]	0.812	0.92 [0.70–1.46]	0.633
Number of ANC visits				
None	1.0		1.0	
1–3	1.41 [0.95–5.01]	0.073	1.19 [0.88–3.96]	0.149
4+	1.52 [1.04–5.58]	0.034	1.54 [1.04–4.91]	0.038
Type of facility where delivery took place				
Hospital	1.0		1.0	
Health center/dispensary	1.06 [0.88–1.55]	0.511	1.02 [0.84–1.32]	0.918
Mode of delivery				
Vaginal	1.0		1.0	
Caesarean section	1.38 [1.05–3.94]	0.039	2.01 [1.38–5.12]	0.002
Cadre of provider attending delivery				
Clinician (doctor)	1.0		1.0	
Midwife or nurse	0.73 [0.48–0.89]	0.004	1.01 [0.38–3.79]	0.952
Others (medical attendant)	0.31 [0.08–0.91]	0.038	1.08 [0.91–1.79]	0.437

aRR: adjusted risk ratio; RR: risk ratio; CI: confidence interval.
